# Chemoradiotherapy versus surgery followed by postoperative radiotherapy in tonsil cancer: Korean Radiation Oncology Group (KROG) study

**DOI:** 10.1186/s12885-017-3571-3

**Published:** 2017-08-30

**Authors:** Sanghyuk Song, Hong-Gyun Wu, Chang Geol Lee, Ki Chang Keum, Mi Sun Kim, Yong Chan Ahn, Dongryul Oh, Hyo Jung Park, Sang-Wook Lee, Geumju Park, Sung Ho Moon, Kwan Ho Cho, Yeon-Sil Kim, Yongkyun Won, Young-Taek Oh, Won-Taek Kim, Jae-Uk Jeong

**Affiliations:** 1 Department of Radiation Oncology, Kangwon National University Hospital, Baengnyeong-ro 156, Chuncheon, 24289 Republic of Korea; 2 Department of Radiation Oncology, Seoul National University College of Medicine, 101 Daehangno, Jongno-gu, Seoul, 110-744 Republic of Korea; 3 Department of Radiation Oncology, Yonsei Cancer Center, 50-1 Yonsei-ro, Seodaemun-gu, Seoul, 03722 Republic of Korea; 4 Department of Radiation Oncology, Samsung Medical Center, Sungkyunkwan University School of Medicine, 81 Irwon-Ro Gangnam-gu, Seoul, 06351 Republic of Korea; 5 Department of Radiation Oncology, Asan Medical Center, University of Ulsan College of Medicine, Seoul, South Korea; 6 Research Institute and Hospital, National Cancer Center, 323 Ilsan-ro, Ilsandong-gu, Goyang-si, Gyeonggi-do 10408 Republic of Korea; 7 Department of Radiation Oncology, Seoul St. Mary’s Hospital, The Catholic University of Korea, 222 Banpo-daero, Seocho-gu, Seoul, 06591 Republic of Korea; 8 Department of Radiation Oncology, Ajou University School of Medicine, Gyeonggi, South Korea; 9 Department of Radiation Oncology, Pusan National University Hospital and Pusan National University School of Medicine, 179 Gudeok-ro, Seo-gu, Busan, 49241 Republic of Korea; 10 Department of Radiation Oncology, Chonnam National University Medical School, 42 Jebong-ro, Dong-gu, Gwangju, 61469 Republic of Korea

**Keywords:** Tonsil cancer, Chemoradiotherapy, Surgery, Adjuvant radiotherapy, Induction chemotherapy

## Abstract

**Background:**

Treatment of tonsil cancer, a subset of oropahryngeal cancer, varies between surgery and radiotherapy. Well-designed studies in tonsil cancer have been rare and it is still controversial which treatment is optimal. This study aimed to assess the outcome and failure patterns in tonsil cancer patients treated with either approaches.

**Methods:**

We retrospectively reviewed medical records of 586 patients with tonsil cancer, treated between 1998 and 2010 at 16 hospitals in Korea. Two hundred and one patients received radiotherapy and chemotherapy (CRT), while 385 patients received surgery followed by radiotherapy and/or chemotherapy (SRT). Compared with the SRT group, patients receiving CRT were older, with more advanced T stage and received higher radiotherapy dose given by intensity modulation techniques. Overall survival (OS), disease-free survival (DFS), locoregional recurrence-free survival (LRRFS), distant metastasis-free survival (DMFS), and clinicopathologic factors were analyzed.

**Results:**

At follow-up, the 5-year OS, DFS, LRRFS and DMFS rates in the CRT group were 82, 78, 89, and 94%, respectively, and in the SRT group were 81, 73, 87, and 89%, respectively. Old age, current smoking, poor performance status, advanced T stage, nodal involvement, and induction chemotherapy were associated with poor OS. Induction chemotherapy had a negative prognostic impact on OS in both treatment groups (*p* = 0.001 and *p* = 0.033 in the CRT and SRT groups, respectively).

**Conclusions:**

In our multicenter, retrospective study of tonsil cancer patients, the combined use of radiotherapy and chemotherapy resulted in comparable oncologic outcome to surgery followed by postoperative radiotherapy, despite higher-risk patients having been treated with the definitive radiotherapy. Induction chemotherapy approaches combined with either surgery or definitive radiotherapy were associated with unfavorable outcomes.

**Electronic supplementary material:**

The online version of this article (10.1186/s12885-017-3571-3) contains supplementary material, which is available to authorized users.

## Background

The tonsils, a subsite of the oropharynx, are the most common site of oropharyngeal neoplasm [1]. The incidence of tonsil cancer is increasing [2, 3]. Odynophagia, dysphagia, otalgia and asymptomatic mass is common presentations. Histologically, squamous cell carcinoma is most commonly observed in tonsil cancer. Regional nodal metastases are frequent in more than half of patients, while contralateral nodal diseases are found in more than one fifth of patients with tonsil cancer [4]. Management of tonsil cancer is limited to either surgery or radiotherapy, yet there is a scarcity of randomized prospective trials comparing these treatment options. However, several retrospective studies published similar oncologic outcomes with both modalities [5–7]. Therefore, current guidelines recommend both strategies based on such findings [8].

In recent decades, breakthroughs in the field have included the introduction of chemotherapy, resulting in improved survival rates after definitive radiotherapy and postoperative radiotherapy [9, 10]. Furthermore, randomized clinical trial data showed that more than half of oropharyngeal cancers were human papillomavirus (HPV) positive and responded well to definitive radiotherapy [11]. The incidence of HPV positive tumors is continuously increasing [12]. In the era of chemotherapy and endemic HPV, comparisons of the efficacy between treatment modalities is still controversial. In the present study, we conducted a large-scale retrospective multicenter study to evaluate the outcome of chemoradiotherapy and surgery followed by postoperative radiotherapy in tonsil cancer patients.

## Methods

A total of 620 tonsil cancer patients who were treated with radiotherapy between 1998 and 2010 were identified in 16 institutions in Korea. Of these, we analyzed data from 586 patients who were treated with definitive radiotherapy with chemotherapy (CRT; 201 patients) or surgery followed by radiotherapy and/or chemotherapy (SRT; 385 patients). All institutional review boards of participating hospitals approved the collection of these data. The need for consent had been waived by the institutional review boards. Patient demographics, performance status, smoking history, imaging study, stage, pathology, type of surgery, radio- and chemotherapeutic information, and follow-up results were compiled.

The median age at diagnosis was 56 (range, 26–89) and patients were predominantly male (89%). The performance status of most patients was Eastern Cooperative Oncology Group (ECOG) grade 0–1 (94%). More than half of the patients (52%) had a history of smoking. Computed tomography (CT) scans of the neck were performed at diagnosis in 91% of individuals; positron emission tomography (PET) or CT scans were taken in 69% of patients, while magnetic resonance imaging of the oropharynx and neck was performed in 48%.

Patient characteristics according to the two treatment groups are summarized in Table 1. Younger patients and those with early T stage were more likely to receive surgery (*p* = 0.041 and 0.002, respectively). Unknown histologic differentiation was less frequent in the SRT group. Chemotherapy and intensity modulated radiotherapy (IMRT) were more commonly used in the CRT group (*p* < 0.001 and 0.014, respectively). Radiotherapy dose was also higher in the CRT group than in those receiving SRT (*p* < 0.001).

**Table 1:** Patient Characteristics

Characteristic	Number of patients (%)						
	All (*n* = 586)		CRT (*n* = 201)		SRT (*n* = 385)		*p*-value
Sex							0.913
Male	523	(89)	179	(89)	344	(89)	
Female	63	(11)	22	(11)	41	(11)	
Age (years)							0.041
< 60	395	(67)	125	(62)	270	(70)	
≥ 60	189	(32)	76	(38)	113	(29)	
Unknown	2	(0)	0	(0)	2	(1)	
Smoker							0.673
Never smoker	232	(40)	73	(36)	159	(41)	
Ex-smoker ^a^	98	(17)	32	(16)	66	(17)	
Current smoker	206	(35)	73	(36)	133	(35)	
Unknown	50	(8)	23	(11)	27	(7)	
Performance							0.351
ECOG 0	197	(34)	74	(37)	123	(32)	
ECOG 1	351	(60)	117	(58)	234	(61)	
ECOG 2	21	(3)	5	(2)	16	(4)	
Unknown	17	(3)	5	(2)	12	(3)	
PET/CT							0.072
No	182	(31)	72	(36)	110	(29)	
Yes	404	(69)	129	(64)	275	(71)	
Differentiation							<0.001
WD	62	(11)	15	(7)	47	(12)	
MD	297	(51)	73	(36)	224	(58)	
PD	129	(22)	37	(18)	92	(24)	
UD	16	(3)	11	(5)	5	(1)	
Unknown	82	(14)	65	(32)	17	(4)	
T stage							0.002
T1	134	(23)	31	(15)	103	(27)	
T2	292	(50)	101	(50)	191	(50)	
T3	74	(13)	30	(15)	44	(11)	
T4a	73	(12)	30	(15)	43	(11)	
T4b	13	(2)	9	(4)	4	(1)	
N stage							0.779
N0	73	(12)	20	(10)	53	(14)	
N1	79	(13)	28	(14)	51	(13)	
N2a	45	(8)	16	(8)	29	(8)	
N2b	307	(52)	105	(52)	202	(52)	
N2c	60	(10)	23	(11)	37	(10)	
N3	22	(4)	9	(4)	13	(3)	
Stage							0.092
I	8	(1)	2	(1)	6	(2)	
II	42	(7)	23	(11)	37	(10)	
III	82	(14)	29	(14)	53	(14)	
IVA	419	(72)	143	(71)	276	(72)	
IVB	35	(6)	18	(9)	17	(4)	
Chemotherapy							<0.001
Induction	61	(10)	33	(16)	28	(7)	
Concurrent	244	(42)	167	(83)	77	(20)	
Adjuvant	13	(2)	1	(1)	12	(3)	
No	268	(46)	0	(0)	268	(70)	
Radiotherapy technique							0.014
3D–CRT	391	(67)	121	(60)	270	(70)	
IMRT	194	(33)	80	(40)	114	(30)	
Unknown	1	(0)	0	(0)	1	(0)	
Total dose of radiotherapy	Median 66		Median 70		Median 63		<0.001
(Gy)	(range, 25.2–76)		(range, 59.4–76)		(range, 25.2–72.6)		

*Abbreviations: CRT* radiotherapy with chemotherapy, *SRT* surgery followed by radiotherapy, *ECOG* Eastern Cooperative Oncology Group, *PET/CT* positron emission tomography/computed tomography, *WD* well differentiated, *MD* moderate differentiation, *PD* poor differentiation, *UD* undifferentiated, *3D–CRT* three-dimensional conformal radiotherapy, *IMRT* intensity modulated radiotherapy

^a^An adult who has smoked at least 100 cigarettes in his or her lifetime but who had quit smoking at the time of diagnosis

Overall survival (OS) was defined as the time from the date of treatment initiation to either death or last follow-up. Disease-free survival (DFS) was defined as the time from treatment initiation to recurrence, death, or last follow-up. Locoregional recurrence-free survival (LRRFS) and distant metastasis-free survival (DMFS) were defined as the time from treatment initiation to locoregional/distant recurrence or last follow-up, respectively. Univariate and multivariate analyses were performed using the log rank test and Cox-proportional hazard regression model, respectively.

## Results

**Fig. 1:**
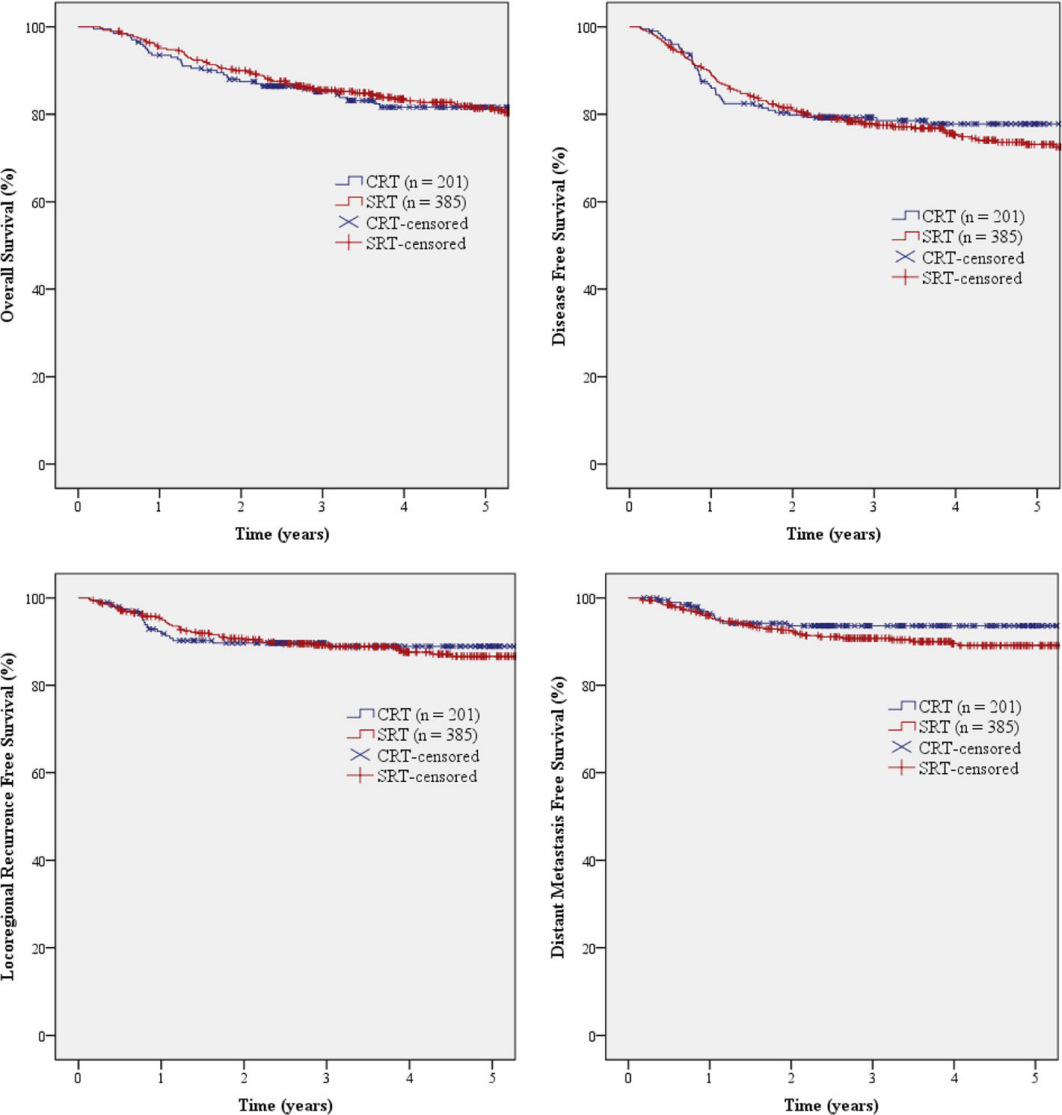
Overall survival, disease-free survival, locoregional recurrence-free survival, and distant metastasis-free survival according to treatment group

With a median follow-up duration of 54 months (range, 2–176 months), 67 (11%) patients demonstrated locoregional recurrence, while 50 (9%) patients failed with distant metastases. The 5-year OS, DFS, LRRFS, and DMFS rates of the cohort as a whole were 81, 75, 87, and 91%, respectively. When the data from the CRT and SRT groups were analyzed independently, no significant differences were observed between the two groups. The 5-year OS rates were 82 and 81% (*p* = 0.698) in the CRT and SRT groups, respectively; DFS, 78 and 73% (*p* = 0.612); LRRFS, 89 and 87% (*p* = 0.695); and DMFS, 94 and 89% (*p* = 0.157). The survival curves of each group are plotted in Fig. 1.

**Fig. 2:**
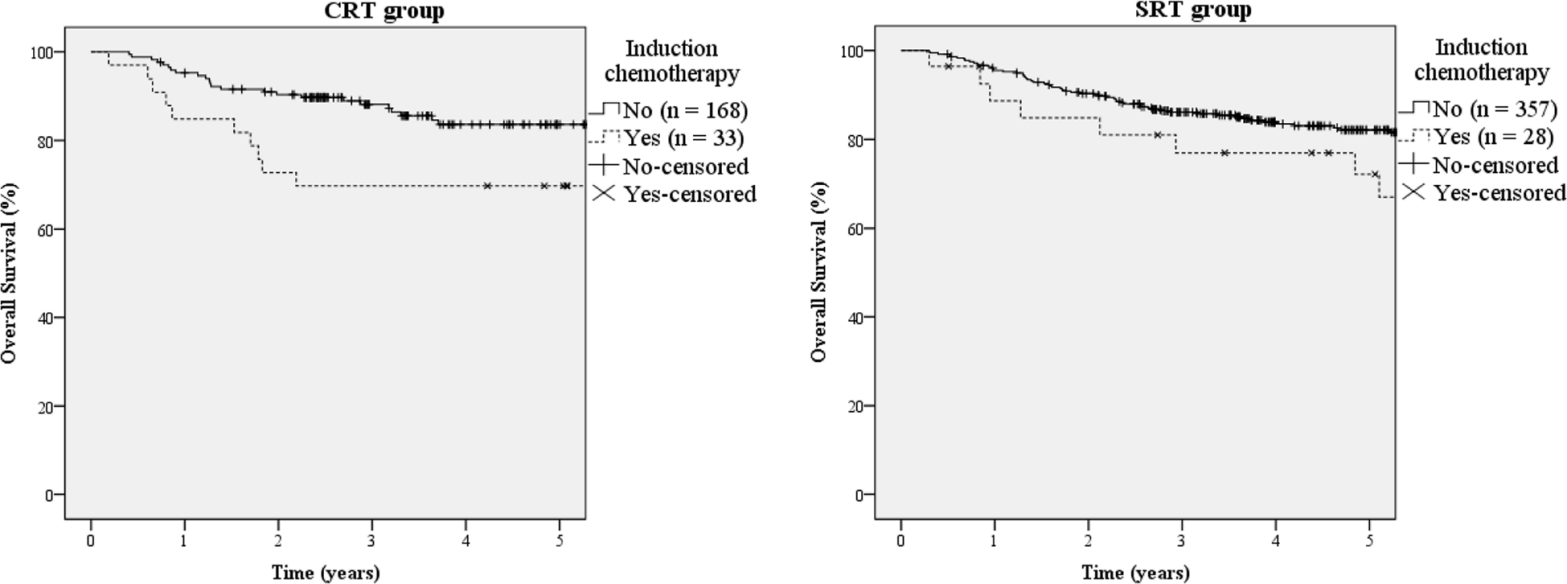
Overall survival according to presence or absence of induction chemotherapy in each treatment group

Older age, current smoking, advanced T and N stage, and induction chemotherapy treatment were associated with poor OS in the univariate analysis (Table 2). Furthermore, patients undergoing induction chemotherapy showed inferior survival in both treatment groups (Fig. 2); the 5-year OS rates of patients treated with and without induction chemotherapy were 71 and 83%, respectively (*p* < 0.001). This significant finding was also observed when the treatment groups were analyzed independently; in the CRT group, the 5-year OS rates of patients with or without induction chemotherapy were 70 and 84% (*p* = 0.001), respectively, and 72% vs 82% in the SRT group (*p* = 0.033).

**Table 2:** Univariate Analyses

Characteristic	OS			DFS			LRRFS			DMFS		
	No.	5Y (%)	*p*-value	No.	5Y (%)	*p*-value	No.	5Y (%)	*p*-value	No.	5Y (%)	*p*-value
Sex												
Male	523	80	0.082	521	73	0.037	525	86	0.025	522	91	0.787
Female	63	90		63	88		63	94		63	91	
Age (years)												
< 60	395	85	<0.001	393	80	<0.001	395	89	0.121	394	92	0.026
≥ 60	189	73		189	62		189	83		189	86	
Smoking history												
Never/ex-smoker	330	85	0.001	329	79	0.001	330	90	0.005	330	92	0.306
Current smoker	206	76		205	67		206	82		205	89	
Performance status												
ECOG 0	197	85	0.094	197	80	0.027	197	93	0.008	197	92	0.45
ECOG 1–2	372	79		370	72		372	85		371	90	
PET/CT												
No	182	79	0.226	181	74	0.847	182	88	0.669	182	93	0.311
Yes	404	83		403	74		404	87		403	89	
T stage												
T1–T2	426	87	<0.001	425	81	<0.001	426	90	0.003	425	94	<0.001
T3–T4	160	67		159	58		160	81		160	82	
N stage												
N0–N2b	504	84	0.005	503	78	<0.001	504	88	0.055	504	83	<0.001
N2c–N3	82	67		81	55		82	81		81	79	
Stage												
I–III	132	90	0.066	132	85	0.016	132	93	0.06	132	97	0.004
IVA–IVB	454	79		452	72		454	86		453	89	
Chemotherapy												
Concurrent/no	525	83	<0.001	523	76	0.006	525	88	0.263	524	90	0.762
Induction	61	71		61	64		61	83		61	93	
Radiotherapy technique												
3D–CRT	391	80	0.420	389	74	0.36	391	87	0.754	390	90	0.235
IMRT	194	84		194	76		194	88		194	92	
Treatment modality												
CRT	201	82	0.698	201	78	0.612	201	89	0.695	201	94	0.157
SRT	385	81		383	73		385	87		384	89	

*Abbreviations: OS* overall survival, *DFS* disease-free survival, *LRRFS* locoregional recurrence-free survival, *DMFS* distant metastasis-free survival, *ECOG* Eastern Cooperative Oncology Group, *PET/CT* positron emission tomography–computed tomography, *3D–CRT* three-dimensional conformal radiotherapy, *IMRT* intensity modulated radiotherapy, *CRT* radiotherapy with chemotherapy, *SRT* surgery followed by radiotherapy

The multivariate analysis (Table 3) also indicated that induction chemotherapy was a risk factor for poor OS and DFS, but not for LRRFS or DMFS. Other prognostic factors such as old age, current smoking, poor initial performance status and advanced T stage were associated with inferior OS. For DFS, advanced N stage was an additional significant prognostic factor. However, in terms of LRRFS, patient age and use of induction chemotherapy were not included in the Cox model. Age, and T and N stage were also identified as independent prognostic factors for DMFS.

**Table 3:** Multivariate Analyses

	OS			DFS			LRRFS			DMFS		
Characteristics	*p*-value	HR	95% CI	*p*-value	HR	95% CI	*p*-value	HR	95% CI	*p*-value	HR	95% CI
Age (years)												
≥ 60	<0.001	3.000	2.001–4.497	<0.001	2.516	1.779–3.560				0.009	2.227	1.217–4.077
Smoking history												
Current smoker	0.012	1.663	1.116–2.478	0.015	1.54	1.089–2.176	0.014	1.919	1.141–3.226			
Performance status												
ECOG 1–2	0.045	1.566	1.010–2.426	0.017	1.587	1.087–2.315	0.019	2.094	1.130–3.879			
T stage												
T3–T4	<0.001	2.913	1.943–4.366	<0.001	2.572	1.808–3.659	0.021	1.852	1.097–3.127	<0.001	3.312	1.804–6.082
N stage												
N2c–N3	0.069	1.542	0.966–2.462	0.008	1.735	1.155–2.607	0.075	1.747	0.946–3.226	0.007	2.435	1.271–4.664
Chemotherapy												
Induction	0.003	2.224	1.313–3.768	0.033	1.712	1.044–2.806						

*Abbreviations: OS* overall survival, *DFS* disease-free survival, *LRRFS* locoregional recurrence-free survival, *DMFS* distant metastasis-free survival, *HR* hazard ratio, *CI* confidence interval, *ECOG* Eastern Cooperative Oncology Group

## Discussion

Controversy surrounds the treatment of tonsil cancer. Both definitive surgery and radiotherapy resulted in favorable outcomes in retrospective studies [5–7]. With the use of chemotherapy, improved survival rates were reported with both treatment modalities [9, 10]. However, no well-designed prospective study comparing radiotherapy and surgery has been completed in the era of widely used chemotherapy. The only prospective randomized trial comparing chemoradiotherapy and surgery followed by radiotherapy was stopped prematurely due to slow accrual and therefore failed to detect any significant difference in DFS between treatment groups [13].

In the present study, we report the outcome of 586 patients from 16 hospitals. To the best of our knowledge, this is one of the largest tonsil cancer cohorts in the literature. We found that old age and advanced T stage which were associated with inferior survival in a multivariate analysis are more found in the CRT group. Despite these discrepancies in patient demographic and disease stage, there was no significant difference between the two treatment modalities under investigation in terms of survival, recurrence, or failure pattern. Our findings suggest that the CRT approach is more effective than SRT; however, further studies are required to confirm this hypothesis.

If the outcome is comparable between two treatment options, morbidity associated with the treatment becomes important when choosing the treatment modality. Unfortunately, we were unable to collect extensive information regarding treatment-related toxicities. Future trials should address not only the outcomes of treatment, but also any associated complications.

The outcomes of our multicenter study are comparable with those of previously published series. Canis et al. reported the outcome of 102 tonsil cancer patients who were treated with transoral laser microsurgery [14]. The 5-year locoregional control rates of T1–T2 and T3–T4 stage tumors were 78 and 75%, respectively. In the current study, the corresponding rates were 90 and 81%, respectively. Similarly, researchers from MD Anderson Cancer Center reported 5-year locoregional control and OS rates of 97 and 86%, respectively, in 120 patients who were treated with tonsillectomy followed by postoperative radiotherapy [15]. Poulsen et al. studied the outcomes of 148 patients who received surgery followed by radiotherapy or definitive radiotherapy [16], yielding 5-year locoregional control and OS rates of 84 and 57%, respectively. Other studies performed before the early 2000s reported lower 5-year locoregional control rates of 63–77% and OS rates of 53–60% [7, 17–19], possibly because these studies included a large proportion of patients who were treated in the pre-chemotherapy era. Although direct comparisons are not possible, our treatment outcomes are acceptable when compared with the literature.

In the present study, the induction chemotherapy approach negatively influenced both OS and DFS, but had little effect on LRRFS or DMFS in the multivariate analysis. These findings suggest that induction chemotherapy may cause non-cancer related death. Recently, randomized trials reported increased toxicities and no survival gain with induction chemotherapy [20, 21]. Despite the limitations of retrospective studies (e.g., patient selection), our findings support the proposal that the toxicity associated with routine use of induction chemotherapy might be potentially harmful to tonsil cancer patients who are highly curable without such treatment. This is further indicated by our finding that patients with tonsil cancer showed favorable prognosis and a low rate of distant metastasis, despite 86% demonstrating stage III–IVA disease.

Tobacco smoking is a well-known risk factor for head and neck cancer [22]. Indian researchers reported that prior tobacco abuse was an independent poor prognostic factor for DFS and locoregional control in oropharyngeal cancer [23]. Less than half of tumors in that study were located in the tonsils. In our study, current smokers showed significantly worse OS, DFS, and LRRFS than non- or ex-smokers in the multivariate analysis. Differing tumor biology in smokers may affect disease outcome [24]. It is well known that persistent smoking during radiation therapy adversely affects the response and survival rate of head and neck cancer patients [25]. Smoking cessation may be beneficial and should be encouraged in patients with tonsil cancer.

Age of >60 years was associated with a significant risk of death, disease recurrence, and distant metastasis in the multivariate analysis. HPV infection, which correlated with favorable prognosis, was more frequently observed in younger patients than in the elderly; [12] therefore, smoking history and old age could be secondary surrogates of poor tumor biology which is unrelated to HPV infection. Unfortunately, because the HPV status of patients in the present study was unknown, this hypothesis could not be tested. Regarding that many recent studies for altering therapy based on HPV status are in progress, the lack of details of HPV status in this study has significant limitations [26].

## Conclusion

Our large, multicenter, retrospective review of tonsil cancer patients showed favorable survival and disease control. Despite more high-risk patients being treated with definitive chemoradiotherapy than surgery followed by radiotherapy, demonstrated comparable outcomes. Furthermore, our study indicated that induction chemotherapy is correlated with significant risk of death and should not be routinely given to tonsil cancer patients.

## Additional file

Additional file 1: Ethics information. (DOCX 13 kb)

## Abbreviations

CRT: Chemoradiotherapy
CT: Computed tomography
DFS: Disease-free survival
DMFS: Distant metastasis-free survival
ECOG: Eastern Cooperative Oncology Group
HPV: Human papillomavirus
IMRT: Intensity modulated radiotherapy
KROG: Korean Radiation Oncology Group
LRRFS: Locoregional recurrence-free survival
OS: Overall survival
PET: Positron emission tomography
SRT: Surgery followed by postoperative radiotherapy
