# Supplementary material for: Chemoradiotherapy versus surgery followed by postoperative radiotherapy in tonsil cancer: Korean Radiation Oncology Group (KROG) study
Source: BMC Cancer. 2017 Aug 30;17:598. doi: 10.1186/s12885-017-3571-3 (PMC5577763; doi:10.1186/s12885-017-3571-3)
Supplement: Additional file 1: — Ethics information. (DOCX 13 kb) [file 12885_2017_3571_MOESM1_ESM.docx]

**Additional file 1**

**Ethics committees**

Ajou University Hosptal, Institutional Review Board

Asan Medical Center, Institutional Review Board

Chonnam National University Hospital, Institutional Review Board

Chung Ang Universtiy Hospital, Institutional Review Board

Ewaha University Medical Center, Institutional Review Board

Gyeongsang National University Hospital, Institutional Review Board

Keimyung University Dongsan Medical Center, Institutional Review Borad

Korea University Guro Hospital, Institutional Review Board

National Cancer Center, Institutional Review Board

Pusan National University Yangsan Hospital, Institutional Review Borad

Samsung Medical Center, Institutional Review Board

Seoul National University Bundang Hospital, Institutional Review Board

Seoul National University College of Medicine/Seoul National University Hospital, Institutional Review Board

Seoul St. Mary’s Hospital, The Catholic University of Korea, Institutional Review Board

Severance Hospital, Institutional Review Board

Yeungnam University Medical Center, Institutional Review Board

**Waiver of informed consent**

Due to retrospective manner of this study, it was not feasible to obtain informed consent. Waiver of informed consent did not seriously affect the validity of the study. There is no reason to estimate the rejection of the subject's consent, and the risk to the subject is extremely low even if the consent is waived. This study was conducted through inquiry of patients' medical records, and did not include intervention such as medication and examination. All data related to the patient's personal information have been kept strictly confidential and protected. Therefore, this study did not infringe the rights of the patients.
